# Pandemic 2009 Influenza A (H1N1) virus infection in cancer and hematopoietic stem cell transplant recipients; a multicenter observational study.

**DOI:** 10.12688/f1000research.5251.2

**Published:** 2015-08-25

**Authors:** Maria Cecilia Dignani, Patricia Costantini, Claudia Salgueira, Rosana Jordán, Graciela Guerrini, Alejandra Valledor, Fabián Herrera, Andrea Nenna, Claudia Mora, Inés Roccia-Rossi, Daniel Stecher, Edith Carbone, Ana Laborde, Ernesto Efron, Javier Altclas, Aníbal Calmaggi, José Cozzi

**Affiliations:** 1 Commission of Infections in Immunocompromised Patients, Argentinean Society of Infectious Diseases (SADI), Buenos Aires, 1085, Argentina; 2 Infectious Diseases, Instituto de Oncología Angel H. Roffo, University of Buenos Aires, Buenos Aires, 1417, Argentina; 3 Infectious Diseases, Sanatorio Anchorena 1425 and Sanatorio Trinidad Mitre, Buenos Aires, 1430, Argentina; 4 Infectious Diseases, Hospital Británico, Buenos Aires, 1280, Argentina; 5 Infectious Diseases, Hospital Rossi, Buenos Aires, 1900, Argentina; 6 Infectious Diseases, Hospital Italiano, Buenos Aires, 1181, Argentina; 7 Infectious Diseases, CEMIC, Buenos Aires, 1425, Argentina; 8 Infectious Diseases, Hospital Municipal de Oncología “Marie Curie”, Buenos Aires, 1405, Argentina; 9 Infectious Diseases, FLENI, Buenos Aires, 1428, Argentina; 10 Hospital San Martín, Buenos Aires, 1900, Argentina; 11 Argentinean Society of Infectious Diseases (SADI), Buenos Aires, 1085, Argentina; 12 Infectious Diseases, Hospital de Clínicas, University of Buenos Aires, Buenos Aires, 1120, Argentina; 13 Infectious Diseases, Hospital Aeronáutico Central, Buenos Aires, 1437, Argentina; 14 Infectious Diseases, FUNDALEU, Buenos Aires, 1114, Argentina; 15 Bone Marrow Transplant, CETRAMOR, Rosario, Pcia. Sta Fé, 2000, Argentina

## Abstract

**Background: **During March 2009 a novel *Influenza A* virus emerged in Mexico. We describe the clinical picture of the pandemic *Influenza A* (H1N1) Influenza in cancer patients during the 2009 influenza season.

**Methods: **Twelve centers participated in a multicenter retrospective observational study of cancer patients with confirmed infection with the 2009 H1N1 *Influenza A* virus (influenza-like illness or pneumonia plus positive PCR for the 2009 H1N1 *Influenza A* virus  in respiratory secretions). Clinical data were obtained by retrospective chart review and analyzed.

**Results: **From May to August 2009, data of 65 patients were collected. Median age was 51 years, 57 % of the patients were female. Most patients (47) had onco-hematological cancers and 18 had solid tumors. Cancer treatment mainly consisted of chemotherapy (46), or stem cell transplantation (SCT) (16). Only 19 of 64 patients had received the 2009 seasonal Influenza vaccine. Clinical presentation included pneumonia (43) and upper respiratory tract infection (22). Forty five of 58 ambulatory patients were admitted. Mechanical ventilation was required in 12 patients (18%). Treatment included oseltamivir monotherapy or in combination with amantadine for a median of 7 days. The global 30-day mortality rate was 18%. All 12 deaths were among the non-vaccinated patients. No deaths were observed among the 19 vaccinated patients. Oxygen saturation <96% at presentation was a predictor of mortality (OR 19.5; 95%CI: 2.28 to 165.9).

**Conclusions:** In our cancer patient population, the pandemic 2009 Influenza A (H1N1) virus was associated with high incidence of pneumonia (66%), and 30-day mortality (18.5%). Saturation <96% was significantly associated with death. No deaths were observed among vaccinated patients.

## Introduction

Seasonal influenza is a known cause of morbidity and mortality among cancer and transplant patients. During influenza season, 20 to 30% of stem cell transplant SCT recipients with respiratory symptoms can test positive for *Influenza* with a mortality rate of up to 28% ^1^. Non-transplant cancer patients can also have a high mortality rate of up to 38% ^2^, being higher in patients with lung, hematological and colorectal cancer, in patients that develop lower respiratory tract infections, and in patients with other co-morbid conditions. In Argentina, seasonal Influenza in onco-hematological patients is associated to a 12% incidence of pneumonia and to a 5% of 30-day mortality ^3^.

In March 2009 a novel *Influenza* A virus, later known as 2009 *pandemic influenza* A (H1N1), emerged in Mexico. The new strain initially spread among travelers to the USA and Canada, and subsequently infected people worldwide ^4^. Clinical presentations ranged from mild symptoms to severe cases that lead to pneumonia and respiratory failure–related deaths.

The first cases of pandemic *Influenza* A (H1N1) in Argentina were reported in May 2009, in travelers returning from Mexico and the USA. From May to December 2009 there were 11931 cases of confirmed *Influenza* A H1N1 in Argentina, 617 deaths, and over 90% of the circulating respiratory viruses in adults were the novel *Influenza* A H1N1 ^5^.

Data from different studies on the impact of this new virus in the adult cancer and SCT population are somewhat contradictory. Many studies from different countries were reported ^1, 6– 16^. In these studies, the incidence of pneumonia ranges from 20 ^11^ to 52% ^8^, while the reported mortality rate ranges from 0–10% ^6, 11, 12, 14, 16^ to as high as 21–31% ^7– 10, 15^.

During the 2013 winter season, pandemic *Influenza A* H1N1 continued to circulate (FluNetDB, WHO, http://apps.who.int/globalatlas/dataQuery/default.asp).

In this study, we examined the effects and severity of *pandemic* H1N1 *Influenza* during the 2009 influenza season, in patients with cancer and SCT in two cities of Argentina.

## Methods

This is a multicenter retrospective observational study that included 12 medical centers. From May to August 2009, cancer and SCT patients older than 16 years, who presented a confirmed influenza infection by real-time PCR were included.

The following data were obtained anonymously: underlying illness, type and date of SCT, whether patients were or were not receiving immunosuppressive treatment, at the time of the influenza diseases, immunization for seasonal influenza, clinical presentation (influenza like or pneumonia), laboratory and radiology results, anti-viral treatment, and outcome. In addition, data on the time between the onset of symptoms and the initiation of antiviral therapy, need for ventilation support, and presence of co-infections were also collected. Hypoxemia was defined as an oxygen saturation value lower than 96%. We diagnosed lymphopenia when the absolute lymphocyte count was less than 1000/µL. The RT-PCR tests for *pandemic Influenza A* H1N1 virus were performed on nasopharyngeal swabs or bronchoalveolar lavage samples when available. Either of two PCR protocols were used for detection of the *pandemic Influenza A* H1N1 virus depending on test availability: the Real-time ready InfluenzaA/H1N1DetectionSet ^®^ Version June 2009 (Roche Diagnostics GmbH, Roche Applied Science68298 Mannheim, Germany) and the PCR protocol used by the WHO (CDC protocol of real-time RTPCR *for influenza A H1N1* 28 April 2009, revision 1, 30 April 2009).

Categorical variables are shown as percentages and they are compared with the χ²-distribution test or Fisher test. Numeric variables such as median and range are compared with the Wilcoxon test. The association between baseline variables and events is presented as OR with the 95% CI. In all cases, statistical significance was assumed at a value of p=<0.05.

## Results

From May to August 2009, 12 centers sent data of 65 cancer patients with 2009 H1N1 virus disease confirmed by positive PCR in BAL (3) or nasopharyngeal wash (62). The median age of the patients was 51 years (range 17 to 81), and 57% were female. The majority of patients (47) had onco-hematological cancer (72%) and 18 (28%) had solid tumors. Cancer treatment included chemotherapy (46), SCT (16), no treatment (2) and surgery (1). History of 2009 seasonal influenza vaccination was present in 19/64 patients (30%). No patient had received influenza chemoprophylaxis. The median time of patients follow up from the onset of symptoms was 61 days, range 5 to 259.

Data on overall clinical presentation and outcome are shown in Table 1. Pneumonia and pneumonia with oxygen saturation <96% were the most common clinical presentations (43/65, 66% and 30/65, 46%, respectively). Co-infections were present in a minority of cases (9/65, 14%) and only among patients with community acquired Influenza.

**Table 1.** Clinical presentation, treatment and outcome of cancer patients and stem cell transplant recipients with confirmed pandemic *Influenza A* H1N1 virus infection.

Clinical presentation	N 65(%)
Upper respiratory infection	22 (34)
Pneumonia - Oxygen saturation <96%	43 (66) - 30/43 (70)
Bacterial co-infections*	7 (11)
Viral co-infections**	2 (3)
Lymphocyte count/uL, median, (range) (N=61)	779 (30-50700)
Neutrophil count/uL, median, (range) (N=63)	3167 (28-68800)
**Treatment and outcome**	
Oseltamivir	56 (86)
Oseltamivir + amantadine	7 (11)
No treatment (patients in terminal phase of underlying disease)	2 (3)
Duration of oseltamivir treatment in days, median (range) (N=63)	7 (1–22)
Required hospital admission (N=58)	45 (78)
Hospital acquired infection	7 (11)
Required ICU admission	17 (26)
Required mechanical ventilation	12 (18)
Length of hospital stay in days, median (range)	8 (1–44)
Days on mechanical ventilation, median (range)	7 (1–42)
Global 30-d mortality	12 (18)
30-day mortality in 19 vaccinated patients	0
30-day mortality in 45 non-vaccinated patients	12 (27)

*4 pneumonias (3 *S. pneumoniae*, 1 *Moraxella catarrhalis*), 3 bacteremia ( *K. pneumoniae,* MRCNS, *Streptococcus* Group C); ** *Influenza B* and *Parainfluenza 3* infection.

Patients started treatment at a median of two days from onset of symptoms (range 0 to 45 days). Sixty eight percent (43/63) of patients started treatment within the 48h after the onset of symptoms. Some patients received combined antiviral treatment because of the potential circulation of seasonal Influenza A H1N1 known to be resistant to oseltamivir ^17^.

**Figure 1.**
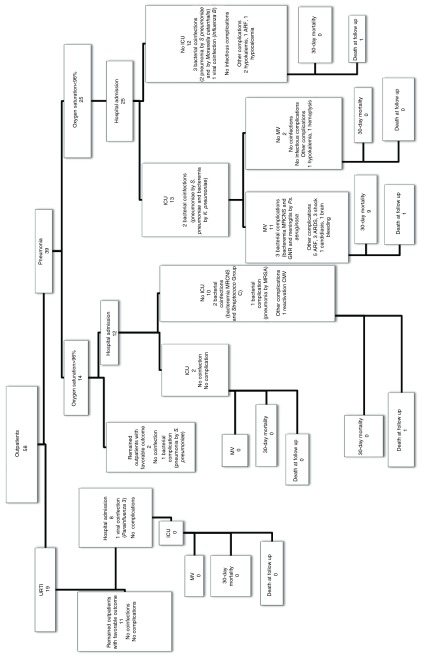
Outcome of immunocompromised patients with community acquired pandemic *Influenza A* H1N1 infection. URTI: upper respiratory tract infection; ICU: Intensive care unit; MV: mechanical ventilation; ARDS: Acute respiratory distress syndrome.

**Figure 2.**
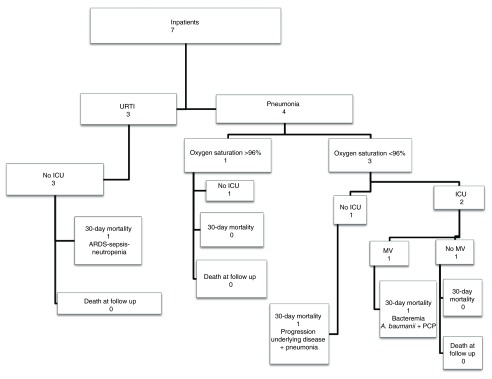
Outcome of immunocompromised patients with nosocomially-acquired pandemic Influenza A H1N1 infection. URTI: upper respiratory tract infection; ICU: Intensive care unit; MV: mechanical ventilation; ARDS: Acute respiratory distress syndrome.

Most patients acquired the infection in the community (58, 89%) while 7 (11%) of infections were acquired in the hospital setting despite the implementation of adequate standard precautions and isolation measures during this outbreak. Detailed descriptions of the outcome of patients with community acquired (CAPIA) and nosocomially-acquired (NAPIA) pandemic Influenza A H1N1 infection patients are described in Figure 1 and Figure 2. The 30-day mortality was higher among patients with NAPIA (3/7, 43%) than among those with CAPIA (9/58, 15,5%).

Most (45/58; 78%) of outpatients required hospital admission. Reasons for patient admission included mainly oxygen desaturation, but, in many cases, patients were admitted because of their severe state of immune suppression and the lack of information about this emergent virus, especially when the patient’s social environment prevented him/her from easy access to medical care.

Outpatients who presented with upper respiratory tract symptoms (URTI), had the most benign course since the majority (11/19, 52%) resolved their infections with antiviral therapy in the outpatient setting, and, among the 8 (42%) who were admitted, none of them required ICU admission or developed signs of pneumonia. The 30-day mortality among CAPIA URTI was 0.

Outpatients who presented with pneumonia had a more severe course since almost all of them (37/39, 96%) were admitted, 15/39 (38%) required ICU, 11/39 (28%) required mechanical ventilation (MV), and the 30-day mortality in this group was of 23% (9/39). The worst prognosis in this group was seen among those who presented with pneumonia and desaturation (25), leading to an admission rate of 100% (52% in ICU, 44% needed MV), and a 30-day mortality of 36%.

Patients, who developed NAPIA, belonged to 3 different centers and started having symptoms at median of 20 days after admission (range 2 to 33). This group had the poorest prognosis since the 30-day mortality rate was 43% (3/7).

One of three (33%) NAPIA URTI progressed to pneumonia, while none of the 19 patients with CAPIA URTI did. Therefore, the overall progression from URTI to pneumonia was of 4.5% (1/22).

The 30-day mortality according to the clinical presentation and setting is best described in Table 2 for comparison. It is shown that having pneumonia at presentation and developing of the infection in the hospital setting tended to be associated with a higher 30-day mortality without achieving statistical significance.

**Table 2.** The mortality of patients with pandemic Influenza A H1N1 infection according to the clinical presentation and setting.

	All URTI	All pneumonias	All CAPIA	All NAPIA	CAPIA pneumonias	CAPIA URTI	NAPIA pneumonias	NAPIA URTI
**30-day** **mortality**	1/22	11/43	9/58	3/7	9/39	0/19	2/4	1/3
**%**	4.5	26	15.5	43	23	0	50	33

URTI: Upper respiratory tract infection; CAPIA: Community-acquired pandemic *Influenza A* infection; NAPIA: Nosocomially acquired pandemic *Influenza A* infection.

Bacterial complications were documented in 6 (9%) patients and included 3 bacteremias (CVC related MRCNS, *Acinetobacter baumanii*, and GNR that was only seen in direct examination), 2 pneumonias (MRSA, *S. pneumoniae*) and 1 meningitis ( *Ps. aeruginosa*). The median time from the onset of symptoms to the development of a bacterial complication was 11 days, range 0–34. Bacterial complications developed only among patients who presented with pneumonia (6/43, 14%) by the pandemic *Influenza A* H1N1. No bacterial complication developed among the 22 patients who presented with URTI.

Non-infectious complications developed in 14 (22%) patients. They included: renal failure (5), respiratory failure (5), shock (3), hypokalemia (3), nonbacterial infections (3) (CMV reactivation, candidiasis by *C. glabrata*, and PCP) and bleeding (2) (lung and brain). Most patients presented more than one complication.

No deaths were observed among patients who had been vaccinated against seasonal influenza in the same year. We could not collect data on the date of vaccination. However, we do know that the first case was detected on May 12 ^th^, while the seasonal influenza vaccine was available since March. Therefore, there is a high probability that at least 14 days might have passed between vaccination and the onset of symptoms.

The presence of any co-infection (bacterial or viral) at onset of symptoms and the delay in treatment were not associated to death or mechanical ventilation. By univariate analysis lack of history of vaccination, and the following baseline characteristics: pneumonia, oxygen saturation <96%, and lymphocyte count <800 cells/μL, were associated to 30-day mortality and mechanical ventilation. By multivariate analysis only lack of history of vaccination (OR did not apply because none died in the vaccinated group) and baseline oxygen saturation <96% (OR 19.5; 95% CI 2.28-165.9; P=0,007) were associated to mechanical ventilation and death. There might be a bias regarding the apparent benefit of vaccination because in cancer patients, immunization is usually advised when the period of major immunosuppression has finished.

Baseline characteristics, clinical presentation, treatment and outcome of 65 cancer or SCT patients Click here for additional data file. Copyright: © 2015 Dignani MC et al. 2015 Data associated with the article are available under the terms of the Creative Commons Zero "No rights reserved" data waiver (CC0 1.0 Public domain dedication).

## Discussion

Our study shows the clinical course of the infection by the 2009 pandemic *Influenza A* H1N1 virus in 65 cancer patients from 12 institutions located in two cities of Argentina. Eleven percent of these infections were nosocomially acquired. Overall we found a high rate of pneumonia (66%) and mortality (18%). The clinical course was less severe in those who presented with an URTI in the outpatient setting in contrast to those who presented with pneumonia and desaturation especially in the hospital setting. We also found that the best predictors of death were oxygen desaturation at presentation and lack of vaccination against seasonal Influenza.

The incidence of pneumonia we found is higher than the one reported with seasonal influenza in cancer patients (5–44%) ^3, 18– 20^ and it is also higher than the incidence of lower respiratory tract infections (LRTI) caused by the 2009 pandemic *Influenza A* H1N1 in the hospitalized general population (40–44%) ^21^, in solid organ transplant recipients (23%) ^16^, in HCT recipients (21–56%) ^1, 6, 8, 12– 14, 22^ and in patients with hematological malignancies (48%) ^23^. Only one small study that includes 15 confirmed cases of 2009 pandemic *Influenza A* H1N1 infection in onco-hematological patients reports a higher incidence of pneumonia (87%) ^15^.

The 30-day mortality rate we show in this study is more than three times the one observed in Argentina in the same patient population when looking at infections by other respiratory viruses such as *Adenovirus, Influenza*, *Parainfluenza*, and RSV (5%) ^3^. However, it is similar to the mortality rate reported in hematological patients with pneumonia by *Influenza*, *Parainfluenza*, *Picornavirus* and RSV at a US institution (15%) ^24^.

It is well known that seasonal influenza-induced pneumonia is independently associated with mortality after HCT (adjusted HR 2.6; 95% CI 1.40-4.86) ^20^. The pandemic *Influenza A* H1N1 virus is an independent risk factor for progression to LRTI (OR 5.64; 95% CI 1.3-25) and hypoxemia (OR 5.91; 95% 1.4–24) compared with seasonal *influenza* virus in HCT recipients ^1^. In addition, immunosuppression was a main risk factor for early mortality among 337 Argentinean patients admitted to ICU with influenza like illness and respiratory failure that required mechanical ventilation. Mortality was highly associated with refractory hypoxemia ^25^. These data explain our high mortality rate observed among the 17 patients who were admitted to ICU (11/17; 65%) or among the 12 patients who developed respiratory failure (11/12; 92%). These values are comparable to those reported in the same type of population ^1, 8, 12, 22^, but are higher than those reported in other populations, which ranged from 0–24% ^21, 26– 29^. Indeed, the overall mortality rate observed among Argentinean patients admitted to ICU and requiring mechanical ventilation was 46% ^25^. To further support the high mortality of patients with pandemic 2009 Influenza pneumonia, we identified hypoxemia at onset of symptoms as an independent predictor of mortality.

Lymphocytopenia has been described as a risk factor for progression from upper to lower viral respiratory tract infection in cancer patients ^24, 30^, and profound lymphopenia (<100 cell/µL) was reported as a significant risk factor for requirement of mechanical ventilation and death in HCT recipients infected with seasonal influenza virus ^30^. In our study, having fewer than 800 lymphocytes/µL at presentation was a predictor for the need for mechanical ventilation and death in a univariate but not in a multivariate analysis. We did not analyze a lower value such as <100 of lymphocytes due to the small number of patients included with this value.

It is noteworthy that co-infections or bacterial complications developed in less than 15% of patients.

Neuraminidase inhibitor therapy appears to be effective in preventing progression to LRTI ^2, 30^ and hypoxemia ^30^ when instituted early after onset of symptoms. It was reported that delaying therapy in cancer patients with the pandemic *Influenza A* H1N1 virus infection was significantly associated with death ^16^. Early initiation of antiviral therapy in these patients may attenuate the severity of disease ^21, 27^. In our series, antiviral therapy was started early after a median of two days after the onset of symptoms, with a range from 0–45 days. We did not find any correlation between days from onset of symptoms to therapy or diagnosis to therapy by univariate or multivariate analysis.

It is known that patients with URTI can be treated as outpatients and can recover completely from their infection ^31^. In our series half of the outpatients with URTI remained as such, while the other half was admitted but did not require ICU. There is a possibility that most of the admitted patients could have been managed as outpatients as well.

The global progression from URTI to pneumonia in our study was of 4.5% (1/22). This single patient had a nosocomial infection and died 21 days later with sepsis, respiratory failure and neutropenia. This is according to reports of progression to LRTI that may occur even after one week of symptoms ^20^.

In contrast, patients with LRTI required hospitalization with a high number of them requiring admission to ICU for ventilation support. The dismal outcome seen in these patients despite treatment with oseltamivir probably indicates that this high-risk group needs to be treated differently from patients with isolated URTI. Some authors have suggested an initial treatment with high dose of oseltamivir and/or combination therapy approaches in the case of respiratory failure ^22^. Higher doses could also be considered in a setting of profound lymphocytopenia ^30^. All our patients received standard dose of oseltamivir (75 mg po twice a day) for a minimum of 10 days based on data on slower viral clearance ^30^.

Nosocomial outbreaks of seasonal ^32^ and pandemic 2009 Influenza A H1N1 infection ^33^ can develop even in the setting of appropriate infection control measures. Seven (11%) of our patients had hospital-acquired influenza. Three of them (43%) died. This mortality rate is higher than the previously reported 13–27%, however, the number of patients in our report is too small to make any conclusion.

Seasonal influenza vaccination is recommended yearly for all patients with cancer and HSCT recipients ^34^. In our study all deaths occurred among the non-vaccinated patients, while there were no deaths among the vaccinated patients. Individuals vaccinated against seasonal Influenza A or with previous seasonal influenza infection may benefit from preexisting cross-reactive memory CD4 ^+^ T cells and CD8 ^+^ T cells reducing their susceptibility *to Influenza A* H1N1 infection or explaining, at least in part, the unexpected mild illness in the community ^35– 39^. Whether the trivalent seasonal Influenza vaccine is protective against the pandemic *Influenza A* H1N1 virus in cancer patients is still a matter of debate ^38^.

In conclusion, we report a series of cancer patients with the pandemic *Influenza A* H1N1 infection with a high incidence of hospitalization, severe pneumonia, ICU admission, mechanical ventilation, and 30-day mortality. In our series hypoxemia and lack of vaccination with seasonal trivalent influenza vaccine were predictors of mechanical ventilation requirement and death. A larger study is needed to evaluate the possibility of cross protection with the seasonal influenza vaccination.

## Data availability

The data referenced by this article are under copyright with the following copyright statement: Copyright: © 2015 Dignani MC et al.

Data associated with the article are available under the terms of the Creative Commons Zero "No rights reserved" data waiver (CC0 1.0 Public domain dedication). 

*F1000Research*: Dataset 1. Baseline characteristics, clinical presentation, treatment and outcome of 65 cancer or SCT patients doi: http://dx.doi.org/10.5256/f1000research.5251.d100276 ^40^

## Consent

Ethical committee approval was not required in Argentina at the time of the study.
