# Supplementary material for: Pandemic 2009 Influenza A (H1N1) virus infection in cancer and hematopoietic stem cell transplant recipients; a multicenter observational study
Source: F1000Res. 2015 Aug 25;3:221. Originally published 2014 Sep 12. [Version 2] doi: 10.12688/f1000research.5251.2 (PMC4240245; doi:10.12688/f1000research.5251.2)
Supplement: Peer review history

# Peer review: Referee response for version 1

**DOI:** 10.5256/f1000research.5598.r6327

**Published:** 2014-11-17

**Authors:** Pablo Bonvehí

**Affiliations:** 1 Infectious Disease Section, Centro de Educación Médica e Investigaciones Clínicas Norberto Quirno (CEMIC), Buenos Aires, Argentina

This is an interesting article that shows the impact of pandemic 2009 influenza A (H1N1) in a highly vulnerable population for influenza complications such as immunocompromised oncohaematological patients.

The paper is well written and I would like to make some comments or questions to the authors:

1. Did you observe any difference in time elapsed since the initiation of symptoms between those with community acquired pandemic 2009 influenza A (H1N1) who presented with upper respiratory tract infections and those who presented with pneumonia? 
2. Previous vaccination against seasonal influenza showed that some degree of preexisting immunity to pandemic 2009 influenza A (H1N1) strains exists, especially among adults aged >60 years. Have you found any difference in progression from UTRI to pneumonia or to death between those older or younger than 69 years of age?

I have read this submission. I believe that I have an appropriate level of expertise to confirm that it is of an acceptable scientific standard.

# Response

**Published:** 2014-12-08

**Authors:** Maria Cecilia Dignani

**Affiliations:** Department of Infectious Diseases, REMIIN (Registro Micosis Invasoras/Registry for Invasive Fungal Infections), Argentina

Question 1:

Patients with community acquired 2009 Influenza A (H1N1) who presented with URTI (N=19) had symptoms for a median of 2 days (range 1-11). Patients who presented with pneumonia (N=39) had symptoms for a median  3 days (range 1-20).

There was a trend towards a longer period with symptoms in patients who presented with pneumonia.

Question 2:

Patients older than 60 years old who had been vaccinated against seasonal Influenza  (N=10) had 60% rate of pneumonia and 0 mortality at day 30 of treatment. Patients with the same age that had not been vaccinated (N=4) had 100% rate of pneumonia and 25% mortality. 

Patients younger than 60 years old that had been vaccinated (N=9) had 55% rate of pneumonia and 0 mortality at day 30 of treatment. Patients with the same age that had not been vaccinated (N=31) had 68% rate of pneumonia and 26% mortality. 

Vaccination against seasonal Influenza seemed to have some degree of protection against death in all age groups.

# Peer review: Referee response for version 1

**DOI:** 10.5256/f1000research.5598.r6116

**Published:** 2014-10-01

**Authors:** Per Ljungman

**Affiliations:** 1 Department of Medicine, Karolinska Institute, Stockholm, Sweden

One of several reports on outcome of the H1N1 pandemic in 2009. A couple of issues:

1. Does the report include all patients fulfilling the inclusion criteria at the 12 hospitals?
2. Why was the cut-off for lymphocytopenia chosen as <800? Most previous studies have chosen a lower cut-off.

I have read this submission. I believe that I have an appropriate level of expertise to confirm that it is of an acceptable scientific standard.
